# Removal of Toxic Metabolites—Chelation: Manganese Disorders

**DOI:** 10.1002/jimd.70107

**Published:** 2025-11-02

**Authors:** Hendrik Vogt, George E. Kostakis, Rupert Purchase, John Spencer, Karin Tuschl

**Affiliations:** ^1^ Department of Genetics and Genomic Medicine, UCL Great Ormond Street Institute of Child Health University College London London UK; ^2^ Department of Chemistry, School of Life Sciences University of Sussex East Sussex UK; ^3^ Sussex Drug Discovery Centre, School of Life Sciences University of Sussex East Sussex UK; ^4^ Department of Analytical, Environmental and Forensic Sciences School of Cancer and Pharmaceutical Sciences, King's College London London UK

**Keywords:** chelation, coordination chemistry, manganese, manganism, SLC30A10, SLC39A14

## Abstract

Manganese (Mn) overload is a characteristic of multiple disease entities, from acquired manganism upon environmental or occupational overexposure, to end‐stage liver disease and certain genetic disorders. The latter include hypermanganesaemia with dystonia 1 and 2 caused by pathogenic variants in the genes encoding the Mn transporters SLC30A10 and SLC39A14. Excess Mn accumulates in the brain, particularly in the globus pallidus, leading to progressive dystonia–parkinsonism. Furthermore, Mn dyshomeostasis is a characteristic feature of common neurodegenerative disorders such as Parkinson's disease, whether as a cause or consequence needs to be determined, suggesting that Mn as an environmental toxicant may play a role in its aetiology. Therefore, there is a need for therapeutics that effectively chelate Mn and remove excess Mn from the brain. This review discusses the Mn chelators currently used in clinical practice, their advantages and disadvantages as well as their adverse effects. Na_2_CaEDTA is the primary chelating agent used to re‐establish Mn homeostasis; however, its burdensome treatment regimen, need for intravenous administration, and lack of metal specificity make it a poor drug for clinical application. The development of novel, Mn‐specific chelating agents is therefore a clinical priority. An ideal chelator would be orally bioavailable, soluble in both lipids and water to reach the sites of metal storage, chemically inert, and non‐toxic whilst retaining chelating abilities at physiological pH. We discuss current progress in identifying novel Mn ligands that have been primarily developed as magnetic resonance imaging contrast agents.

## 1 Introduction

### 1.1 Manganese Physiology

Manganese (Mn) is an essential trace metal that is required for normal cellular physiology and organ development [1]. Mn is ubiquitously found in the environment and acquired via dietary ingestion. It plays a crucial role as an enzyme cofactor in amino acid, lipid, protein, and carbohydrate metabolism [2, 3]. This is evident in the number of Mn‐dependent enzymatic families that include oxidoreductases, transferases, lyases, isomerases, hydrolases, and ligases, as well as the Mn metalloenzymes arginase, glutamine synthetase, phosphoenolpyruvate decarboxylase, and Mn superoxide dismutase (SOD) [4]. Thus, Mn is essential for the maintenance of cellular energy levels, immune response, antioxidation, digestion, blood clotting, blood‐sugar levels, and connective tissue growth [5]. It is also vital for neurotransmitter synthesis and, therefore, critical for neuron and glial cell function [2, 6, 7].

Among its possible oxidation states (ranging from −III to +VII), the divalent Mn(II) (=Mn^2+^) and trivalent Mn(III) (=Mn^3+^) ions are the most prevalent in human tissues [8, 9]. Mn(II) is a critical component of the human diet. In biological environments, Mn can be present as the Mn(II) ion (bound to endogenous low‐molecular weight ligands—mainly citrate) and Mn(III). Above pH 2 in aqueous solutions, Mn(III) disproportionates to Mn(II) and Mn(IV) (precipitated as MnO_2_) unless stabilized by coordination by a ligand [10]. The Mn(II) ion is stable in aqueous solutions up to pH *ca*. 7.5. In this review, Mn refers to the oxidation state Mn(II) unless otherwise stated. In the bloodstream of animals, Mn(II) can be oxidized to Mn(III) on binding to transferrin (Tf) and transported as Tf‐Mn(III) into cells where it reverts to Mn(II) [1, 10].

During adulthood, the estimated average requirement (EAR) of Mn is 2.3 and 1.8 mg/day for men and women, respectively [11]. Mn requirements increase during lactation and gestation. During early childhood development, the Mn requirement increases from 3 μg/day (< 6 months old) to 600 μg/day (between 7 and 12 months) [11]. The EAR of Mn is 1.2 mg/day between 1 and 3 years and 1.5 mg/day between 4 and 8 years [4, 11]. Due to its ubiquitous presence in the diet, nutritional Mn deficiency is not usually reported in the clinic [4].

### 1.2 Regulation of Mn Homeostasis

Mn homeostasis is tightly regulated through several mechanisms, which can be divided into three interwoven categories—absorption, excretion, and cross‐cellular transport. It is estimated that 1%–5% of ingested Mn is absorbed via the gastrointestinal system [12]. Additionally, Mn can also be transported via the pulmonary epithelium and the olfactory nerve after inhalation during environmental and occupational exposure [13, 14, 15]. The amount of gastrointestinal Mn absorption differs by sex, with women absorbing significantly more Mn from the diet than men, likely caused by the lower iron and ferritin concentrations in females. Mn and Fe compete for the same gastrointestinal transporters such as the Divalent Metal Transporter 1 (DMT1), resulting in competitive inhibition of transport between both metals (see Section 1.3.1) [11, 16, 17].

Following gastrointestinal absorption, Mn enters the portal circulation primarily in the form of Mn(II) (> 99%) that binds to alpha‐2‐macroglobulin or albumin. A small amount of Mn is bound to Tf in the form of Mn(III) [18]. In the bloodstream, erythrocytes contain > 60% of Mn while leukocytes and platelets account for 30%; plasma contains only 4% [19]. Blood Mn concentration in adults ranges between 4 and 15 μg/L. [20] Once in the portal circulation, Mn enters the liver, the main regulator of Mn homeostasis, facilitating biliary excretion of Mn and elimination of Mn in the feces [21]. Amongst various tissues, liver, kidney, and pancreas store the highest amounts of Mn. If Mn homeostasis fails and systemic Mn levels rise, Mn accumulates in the brain, the critical target organ of manganese toxicity. Within the cell, Mn is mainly found in the mitochondria and the nucleus [18].

While a number of transporter proteins have previously been implicated in Mn trafficking, recent advances in our understanding of Mn biology suggest that the three solute carriers (SLC), SLC39A8 (ZIP8), SLC39A14 (ZIP14), and SLC30A10 (ZnT10) are the main regulators of Mn homeostasis (Table 1) [6, 26, 27]. Pathogenic variants in the encoding genes lead to inherited disorders of Mn transport characterized by Mn dyshomeostasis [28]. These transporters are part of the Zrt‐ and Irt‐like family proteins (ZIP) and zinc (Zn) transporter protein family (ZnT), respectively [29].

**TABLE 1:** Mn transporters linked to monogenic human diseases.

Transporter protein	Metal substrate	Role	Expression	Disease (OMIM)	References
SLC30A10	Mn (Zn)	Exporter at the apical membrane facilitating Mn excretion.	Brain, GI tract, liver, bone marrow and lymphoid tissue	HMNDYT1 (# 613280); Mn overload	[22]
SLC39A8	Mn (Zn, Fe, and Cd)	Importer at the apical membrane facilitating Mn uptake.	Ubiquitous, high expression in brain, lungs, kidney, GI tract, liver, bone marrow and lymphoid tissue	CDG2N (# 616721) Mn deficiency	[23]
SLC39A14	Mn (Zn, Fe, Cd)	Importer at the basolateral membrane facilitating Mn uptake followed by excretion via SLC30A10.	Ubiquitous, high in GI tract, liver, kidney, bone marrow and lymphoid tissue	HMNDYT2 (# 617013) Mn overload	[24]

*Note:* Biallelic, loss‐of‐function variants in the encoding genes lead to Mn dysregulation in humans. Alternative metal substrates to Mn are given in parentheses. Expression stated as per The Human Protein Atlas (https://www.proteinatlas.org/). Table inspired by Liu et al. [25].

Abbreviations: Cd, cadmium; CDG2N, congenital disorder of glycosylation type IIn; Fe, iron; HMNDYT1, hypermanganesaemia with dystonia 1; HMNDYT2, hypermanganesaemia with dystonia 2; Zn, zinc.

**FIGURE 1:**
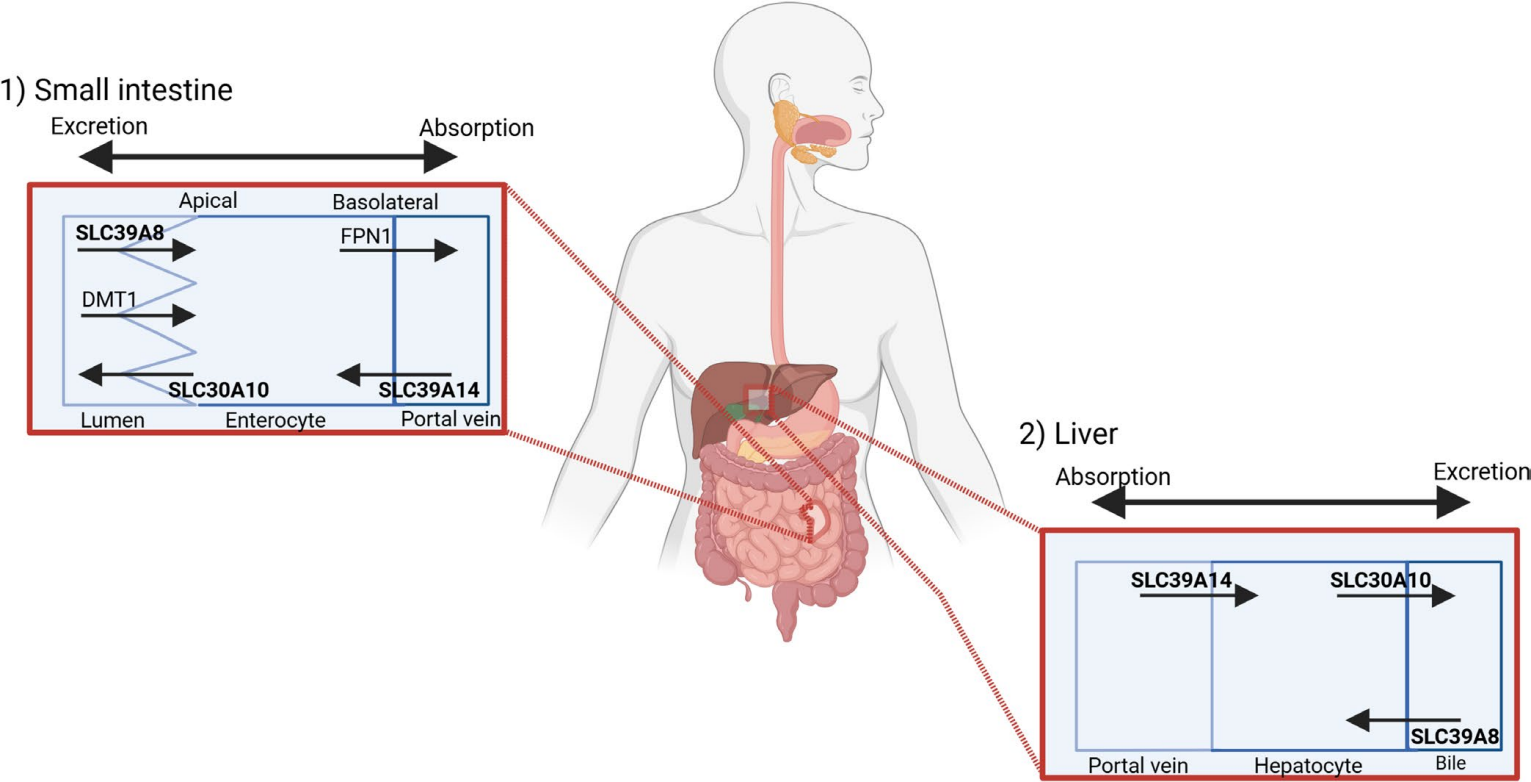
Schematic depicting the transport of Mn following ingestion in the small intestine and liver along with the key Mn transporters. Schematic created with BioRender.

In the small intestine, Mn is mainly absorbed via the duodenum and jejunum [30, 31]. Mn is supposedly taken up into enterocytes at the apical membrane via SLC39A8 and excreted into the bloodstream basolaterally, most likely via ferroportin (FPN1) (Figure 1) [32, 33, 34].

The identification of biallelic, pathogenic variants in *SLC39A8* in patients with inherited, profound Mn deficiency supports the hypothesis that SLC39A8 is the main Mn transporter required for intestinal uptake [26]. Indeed, intestinal Slc39a8 knockout (KO) mice are deficient in Mn in the blood, enterocytes, and other organs without significant changes in Zn and Fe concentrations [32]. SLC39A8 is also expressed on the apical canalicular membrane of hepatocytes; thus, acting to reabsorb Mn from the bile [23]. Consistent with this finding, overexpression of liver‐specific human SLC39A8 leads to elevated levels of Mn in various tissues and blood [23]. Alternative intestinal uptake may also occur via DMT1; however, DMT1 KO in rodents, while affecting Fe homeostasis, does not lead to changes in Mn levels [34, 35].

Localized on the basolateral membrane of enterocytes and hepatocytes, the Mn influx transporter SLC39A14 shuttles Mn from the blood into the enterocyte and hepatocyte for export via SLC30A10 allowing excretion in feces and bile (Figure 1) [36]. Mice lacking intestinal Slc39a14 show impaired basolateral to apical secretion of Mn and increased levels of Mn in the liver and brain. Similarly, liver‐specific Slc39a14 KO mice develop a decrease in hepatic Mn content [36].

SLC30A10 is the crucial exporter of Mn and essential for the regulation of Mn homeostasis [27]. Localized on the apical side of enterocytes and hepatocytes, SLC30A10 exports Mn into the intestinal lumen and the bile for excretion (Figure 1) [25, 37]. Slc30a10 endoderm specific KO mice with absent SLC30A10 expression in the liver and gastrointestinal tract develop highly elevated levels of Mn in the blood, brain, and liver [37, 38].

More recently, SLC39A11 has been suggested to play a role in Mn homeostasis in both mouse and zebra fish, with loss‐of‐function resulting in increased blood Mn levels [39]. However, no human disease has been associated with this gene to date.

### 1.3 Shared Roles of Mn Transporters With Transition and Other Metals

#### 1.3.1 Mn and Fe

Many parallels can be drawn between the chemical and structural properties of Mn and Fe; for example, their atomic and ionic radii, the existence of multiple oxidation states (−III to +VII for Mn, −II to +VI for Fe), preference for octahedral coordination geometry at low oxidation states, and ionization energies [40]. Similarities also extend to the shared use of transporters. DMT1 (SLC11A2) is one such transporter that can transport Mn, Fe, Zn, copper (Cu), and calcium (Ca) along with other metals [41, 42, 43]. It was previously thought that DMT1 was the main importer of Mn via the intestine [41, 42]. However, intestinal DMT1 KO mice do not require DMT1 for the absorption of Mn [35]. Tf‐Mn(III) can further enter the cell via the transferrin receptor (TfR). When vesicles undergo acidification, Mn(III) ions, which have been released by the Tf‐TfR complex, are reduced to Mn(II) ions, allowing them to be transported out of the endosome by DMT1 [44]. Mn and Fe compete for both DMT1 and TfR, contributing to Fe and Mn levels having an inverse relationship. Consequently, blood Mn levels are increased in individuals with iron deficiency, while iron overload reduces Mn uptake [45, 46]. This is corroborated by studies in rats where a diet high in Mn leads to lower Fe levels in plasma and the brain compared to controls [47]. This interdependency of transport is taken advantage of in patients with mutations in SLC30A10 who present with depleted iron stores and are treated with Fe supplements along with other treatment strategies to lower systemic levels of Mn [48].

It is speculated that FPN1, localized on the basolateral side of enterocytes, allows the transport of Mn from the enterocyte into the portal vein [34, 49]. The ability of FPN1 to transport Mn has also been demonstrated in *Xenopus laevis* oocytes expressing human FPN1, human embryonic kidney (HEK) 293 cells, and in mouse brains [34, 50, 51, 52].

#### 1.3.2 Mn and Zn

SLC39A8 transports multiple cations including Mn, Zn, Fe, and cadmium (Cd) [53, 54, 55]. In agreement with this finding, Fe and Zn uptake from the culture medium is increased upon SLC39A8 overexpression in HEK293 cells [56]. Despite this, Mn appears to be the main metal substrate of SLC39A8 since humans with biallelic pathogenic variants in *SLC39A8* present primarily with Mn deficiency [26, 57]. Accordingly, inducible *Slc39a8* KO mice develop systemic Mn deficiency with Mn content being reduced in the liver, brain, heart, and kidney [23]. Observations in *Xenopus* oocytes further suggest that Mn is a potent competitor of Slc39a8‐mediated Zn uptake [58].

As for SLC39A8, SLC39A14 was originally thought to be responsible for Zn uptake. However, overexpression studies confirmed that SLC39A14 can also transport Mn, Fe and Cd [59, 60, 61, 62]. The identification of patients with mutations in SLC39A14 that result in Mn overload and early‐onset dystonia highlights SLC39A14's crucial role in Mn transport [6, 63].

Similarly, SLC30A10 was categorized into the Znt family based on its amino acid sequence that is similar to that of the Zn efflux transporter, ZnT1 [64, 65]. The identification of patients with mutations in SLC30A10 who develop systemic Mn overload provided new insights into the function of SLC30A10 as a primary Mn efflux transporter [27]. Work in chicken DT40 cells suggests that the change in metal specificity between ZnT10 and ZnT1 is due to a histidine residue in position 43 (H43N), thus changing its character from hydrogen bonding (CONH_2_) to metal binding (pyrrole), which is required for the Zn coordination site in ZnT1 and other ZnT transporters [66].

#### 1.3.3 Mn and Ca

Mn(II) and Ca(II) have comparable ionic radii and charges, allowing transport via shared transporters [67]. This is demonstrated at store‐operated Ca^2+^ channels, ionotropic glutamate receptors, DMT1, and the mitochondrial Ca^2+^ uniporter (MICU1) [44, 68, 69, 70, 71, 72, 73]. It has also been shown that Ca^2+^ channel blockers, such as vanadate and nifedipine, reduce the uptake of Mn into erythrocytes, thus, demonstrating that Mn can utilize Ca transporters and channels [74, 75].

### 1.4 Clinical Relevance of Mn Neurotoxicity

#### 1.4.1 Mechanisms Underlying Mn Neurotoxicity

Despite Mn's role as an essential trace metal and enzyme co‐factor, overexposure to Mn is neurotoxic. Excess Mn preferentially accumulates in the basal ganglia, particularly, the globus pallidus, and causes a Parkinsonian movement disorder, termed “manganism”, that is associated with psychiatric and cognitive impairments [76, 77]. This is corroborated by x‐ray fluorescence (XRF) imaging in a Mn exposed rat model that demonstrates Mn accumulation within the globus pallidus and substantia nigra pars compacta [78, 79]. Mn is taken up into the brain by active transport at the blood–brain barrier as well as the blood‐cerebrospinal fluid (CSF) barrier. Mn is transported into the brain either as Mn(II), for example, in the form of Mn(II) citrate complexes, or Tf‐Mn(III). All the transport mechanisms known to date play a part in delivering Mn to the brain, and it is unlikely that Mn influx across the blood–brain barrier can be attributed to a single carrier [80, 81]. It is believed that Mn is cleared from the brain predominantly by slow diffusion. Under conditions of excessive Mn exposure, the rate of efflux falls below the rate of uptake, resulting in the accumulation of Mn in neural tissues [82]. Mn exists as both Mn(II) and Mn(III) in the brain with the latter valency being less stable and a potent oxidizing agent, especially in the mitochondrial environment [83, 84, 85]. There is ongoing debate regarding how Mn is distributed within neuronal subcellular compartments. While some studies suggest that Mn accumulates intracellularly within neuronal mitochondria and lysosomes of the basal ganglia, XRF imaging of dopaminergic neurons has demonstrated Mn deposition in the cytoplasm, particularly with a perinuclear distribution [1, 78, 84, 86, 87, 88, 89]. Mn can also be deposited extracellularly. This can aggravate inflammation and neuronal death as observed in neurodegenerative diseases such as Parkinson's disease (PD) [90].

Mn deposition induces multiple mechanisms of neurotoxicity that include oxidative stress, mitochondrial dysfunction, Ca dysregulation, neuroinflammation, disruption of neurotransmitter signalling, impaired protein handling, and secondary iron deposition [87, 91, 92, 93, 94]. Mn has been implicated in aggravating oxidative stress and in the production of reactive oxygen species (ROS). ROS such as hydrogen peroxide, hydroxyl radical and superoxide radical elicit damages to nucleic acids and phospholipids [87, 95]. As alluded in Section 1.3.3, Mn(II) is taken up into mitochondria via MICU1 and resides in Ca^2+^ binding sites causing Ca dysregulation [96]. The disruption of Ca homeostasis along with induced oxidative stress causes a mitochondrial permeability transition. This leads to loss of mitochondrial inner membrane potential, dysregulated oxidative phosphorylation, impaired ATP synthesis and ultrastructure swelling. These factors culminate in apoptosis and neurodegeneration [87, 95, 97, 98].

Mn has also been heavily implicated in impairing protein folding, trafficking and homeostasis. Therefore, Mn neurotoxicity has been considered as a contributiong factor in the development of neurodegenerative diseases such as PD and Alzheimer's disease (AD) [99, 100]. Mn bound to alpha‐synuclein (αSyn) induces conformational changes that lead to αSyn aggregation [101, 102, 103, 104]. Overexpression of αSyn leads to elevated levels of cellular Mn, independent of relevant transport genes, which implicates αSyn's role in storing Mn [105]. Dopaminergic neuronal death has also been suggested to occur due to Mn‐induced dopamine autooxidation [106]. However, differences exist between manganism and PD. Patients with manganism develop a characteristic movement disorder type that is distinct from PD and lack response to levodopa therapy. Dopaminergic neurons within the substantia nigra are preserved in patients with manganism [107]. Recent evidence suggests that Mn impairs dopaminergic neurotransmitter release rather than causing dopaminergic neurodegeneration. This may be linked to impaired synaptic vesicle fusion upon Mn exposure [108, 109].

Similarly, Mn was shown to upregulate Amyloid β (Aβ) accumulation and was thus suggested as a risk factor of AD [110]. Although Mn does directly interact with Aβ it is likely that Mn modulates amyloidogenesis as opposed to directly inducing amyloid pathology [111, 112, 113, 114]. Following Mn exposure, Tau protein, a hallmark of AD, was correlated to the demethylation of a key tau phosphatase, protein phosphatase 2A (PP2A) [100, 115]. Preventing PP2A demethylation improves cell viability associated with lower pTau levels, oxidative stress and apoptosis [100, 115, 116].

In addition to altered dopaminergic neurotransmission, Mn has also been reported to affect norepinephric, serotonergic, cholinergic, glutamatergic and GABAergic neurotransmission [106, 117, 118, 119, 120, 121, 122, 123, 124, 125, 126].

#### 1.4.2 Acquired Manganism Through Mn Overexposure

Occupational overexposure to Mn, for instance in mining and welding industries, has long been known to lead to increased levels of Mn in the globus pallidus, along with motor symptoms such as bradykinesia, dystonia and akinetic rigidity, and psychiatric disturbances. All of these are hallmark features of “manganism”, a “Parkinson's‐like” movement disorder [127]. Acquired manganism can also occur upon environmental toxicity via contaminated drinking water or prolonged total parenteral nutrition containing more than the recommended amounts of Mn and associated with liver dysfunction [128, 129, 130]. Furthermore, manganism has been described in ephedrone (methcathinone; 2‐methylamino‐1‐phenyl‐1‐propanone) drug addicts due to the use of potassium permanganate in its synthesis [131]. Intravenous administration of Mn in particular increases the risk of toxicity since it circumnavigates the gastrointestinal tract and the liver's homeostatic mechanisms to regulate Mn levels, thus leading to Mn neurotoxicity [130]. Acquired manganism is rare and its exact incidence not precisely defined. However, in occupational risk groups such as welders and miners 15%–30% of chronically exposed workers develop subclinical neurological signs including mild motor and cognitive defects [132, 133, 134].

#### 1.4.3 Acquired Hepatocerebral Degeneration—Manganism due to Liver Cirrhosis

In chronic liver disease, where biliary excretion of Mn (as well as other metals and metabolites such as ammonia) is impaired, and in porto‐systemic shunting, systemic Mn levels rise with increased deposition of Mn in the brain [135, 136]. More than 60% of patients with cirrhosis have elevated blood Mn concentrations, and over 80% demonstrate T1‐weighted hyperintensity of the basal ganglia on MRI, reflecting Mn accumulation [137]. Thus, patients can present with a neuropsychiatric movement disorder manifesting as ataxia, tremor, chorea, dysarthria and parkinsonism as well as cognitive and visuo‐spatial impairments [135, 138, 139, 140, 141].

#### 1.4.4 Inherited Mn Transporter Defects

The identification of the inherited Mn transporter defects, leading to symptoms of manganism during early childhood, has dramatically improved our understanding of Mn biology and its regulation in humans [6, 27, 142]. Biallelic loss‐of‐function variants in the genes encoding the two Mn transporters, SLC30A10 and SLC39A14, cause autosomal recessive movement disorders termed hypermanganesemia with dystonia 1 (HMNDYT1) and 2 (HMNDYT2), respectively. Since SLC30A10 and SLC39A14 act in conjunction to lower the body's Mn load, both disorders lead to increased levels of Mn in the blood and brain associated with neurotoxicity [143, 144]. To date, over 100 patients have been diagnosed worldwide [145]. Most affected patients present during early childhood with generalized dystonia, dysarthria, bradykinesia, and hypomimia. Typically, whole‐blood Mn levels are raised above 2000 nmol/L (normal values < 320 nmol/L). Acquired cases of hypermanganesemia more commonly display Mn levels < 2000 nmol/L. [143] Magnetic resonance imaging (MRI) of the brain is characteristic of Mn deposition with hyperintensity of the basal ganglia, namely the globus pallidus, putamen, subthalamic, dentate, and caudate nuclei, on T1‐weighted imaging [146, 147, 148, 149].

Attenuated forms with predominant adult presentation have been described for both HMNDYT1 and HMNDYT2. Two siblings with HMNDYT1 presented in their 40s and 50s with Parkinsonism, namely rigidity, shuffling gait, hypomimia, bradykinesia, and monotone speech [27]. A female patient was diagnosed with HMNDYT2 at the age of 65 years, who presented with longstanding dystonia, dysarthria, and bradykinesia [148].

In addition to its neurological presentation, HMNDYT1 also leads to non‐neurological symptoms including polycythaemia, depleted iron stores, and chronic liver disease due to deposition of Mn in the liver, which is absent in HMNDYT2. Increase in hepatic Mn inhibits prolyl hydroxylation of hypoxia‐inducible factors (HIFs), which otherwise target HIFs for degradation [150, 151]. Subsequent transcriptional upregulation of erythropoietin (EPO) expression drives polycythaemia in patients with HMNDYT1 [150]. Patients with HMNDYT1 can present with isolated or predominant polycythaemia [152]. Hepatic Mn deposition further causes fibrosis, steatosis, and micronodular cirrhosis, as well as depletion of iron stores [143].

## 2 Chelation Therapy

### 2.1 Principles of Chelation Therapy

Sir Gilbert Morgan (1870–1940) coined the word chelation since the coordination of a bidentate ligand to a metal ion resembles a crab's claw (Greek *chēlē* (*χηλή*)), which saw the emergence of chelation therapy in medicine, the removal of toxic or unwanted metal ions in vivo by a chelating agent [153].

Targeting one specific metal can be challenging due to the presence of other competing metals, accessibility of the target tissue to the chelating agent, and the strength of the metal‐chelating agent binding [154]. Chelating agents enable the mobilization of the metal from the target tissue and subsequent excretion through bile and urine. A more water‐soluble chelating agent allows for a higher degree of transport in the blood and excretion through the kidneys as well as oral delivery. A chelating agent that is more lipophilic may allow for a greater transport of the chelating agent across the cell membranes and the BBB [155]. An ideal chelating agent would be highly soluble in both lipids and water to reach the sites of metal storage, chemically inert, and non‐toxic whilst retaining chelating abilities at physiological pH [156, 157]. Most chelating agents used in clinical practice are not able to cross the blood–brain barrier and therefore have limited ability to remove metals from the brain [155].

### 2.2 History of Metal Chelation in Medicine

British anti‐Lewisite, BAL (2,3‐dimercaptopropanol) was the first chelating agent with a role in medicine. Developed during the Second World War as an antidote to the warfare gas Lewisite [dichloro(2‐chlorovinyl)arsine], BAL can chelate metalloids (arsenic (As)) and heavy metals and was the first chelating agent used to treat the Cu storage disorder Wilson's disease (WD) [158]. Na_2_CaEDTA was used in the 1950s to treat lead (Pb) intoxication, followed by the development of D‐penicillamine, the first oral drug for Wilson's disease, deferoxamine and deferiprone to reduce Fe overload, and dimercaptosuccinic acid (DMSA) (a BAL analog) for the treatment of As and mercury (Hg) poisoning [158, 159, 160, 161, 162].

### 2.3 Current Treatment of Mn Overload

Reducing the body's Mn load alleviates manganism‐associated symptoms in patients with Mn overload conditions. This is correlated with improvements in brain MRI appearances that show reduced T1‐hyperintensity [27, 107, 146]. Lowering Mn levels is mainly achieved through chelation therapy that facilitates metal binding and subsequent urinary excretion. Jean Rodier suggested the use of an EDTA salt for the treatment of Mn toxicity observed in Moroccan miners [163, 164]. Current clinical practice uses disodium calcium edetate (Na_2_CaEDTA) as the chelating agent of choice for Mn overload [145]. Patients with HMNDYT1 and HMNDYT2 have been successfully treated with Na_2_CaEDTA evidenced by the improvement of neurological symptoms and reduction of Mn deposition on brain MRI [5, 48, 107, 145, 146, 165, 166]. However, response to chelation therapy can be varied, particularly in HMNDYT2, with some patients deteriorating upon treatment initiation [6, 27, 147, 149, 165, 167, 168]. This may be due to other roles of SLC39A14 as an uptake transporter in HMNDYT2. There is evidence from studies in zebrafish that Slc39a14 loss‐of‐function may also lead to a deficiency of Mn in distinct subcellular regions or cell types, in addition to systemic Mn overload [142]. Hence, chelation may lower Mn levels further in deficient regions with worsening outcome [6, 142, 169]. Other chelating agents have been trialled in Mn overload with varied clinical responses (discussed below) [27, 170, 171, 172, 173, 174, 175, 176, 177].

In addition to chelation therapy, Fe can lower the body's Mn load since it is a competitive inhibitor of Mn influx in the intestine [40, 178]. This is particularly evident in patients with HMNDYT1 who present with depleted Fe stores. Fe supplementation can lead to a reduction in blood Mn levels, resolution of polycythaemia, and amelioration of neurological sequelae, either in conjunction with Na_2_CaEDTA chelation therapy or on its own [27, 179].

While chelation therapy with Na_2_CaEDTA shows some definite effect on clinical symptoms, associated adverse effects and a burdensome treatment regimen make Na_2_CaEDTA a poor chelating agent for clinical application [6, 27, 143, 144, 165]. Na_2_CaEDTA is not orally bioavailable and thus requires intravenous administration. Long‐term venous access is both invasive and associated with a risk of serious infections. Monthly treatment cycles of five consecutive days with two infusions per day require repeat hospital admissions that interfere with schooling and employment. High socio‐economic costs limit patient access to treatment, particularly, since many patients with inherited Mn transporter defects are from low‐income countries where consanguineous marriages are common [143, 144]. Adverse effects of Na_2_CaEDTA include nephrotoxicity and bone marrow suppression [143, 144, 180]. Furthermore, non‐specific chelation of other metals, such as Ca and Zn, leading to spinal fractures and anaemia, may require additional treatment with for example, Zn supplementation [143, 144].

Children affected by HMNDYT1 and HMNDYT2 commonly present within the first years of life, sometimes as early as 8–12 months of age. Initiating treatment before the onset of symptoms is expected to improve clinical outcomes by preventing Mn accumulation; therefore, early diagnosis is critical [145]. The advent of genetic newborn screening offers a unique opportunity to identify affected infants before clinical symptoms develop [181, 182]. HMNDYT1 and HMNDYT2 are well‐suited for inclusion in genetic newborn screening programs, as their asymptomatic period in infancy provides a crucial window for early intervention. Consequently, there is an urgent need to develop safe chelating agents that are Mn‐specific, more easily administered (e.g., orally bioavailable), and capable of minimizing adverse effects. Such treatments should effectively reduce symptoms, prevent mortality, and significantly improve the quality of life for affected patients.

## 3 Chelation and the Mn(II) Ion

Limiting access of Mn to the blood–brain barrier by chelation of blood‐bound Mn(II) is the primary therapeutic option for ameliorating the neurotoxic effects of Mn overload. Effective chelation of Mn requires ligands that produce kinetically inert Mn complexes with high stability constants and are selective for Mn in the presence of other metals (e.g., Zn(II) in a biological environment). However, compared with the coordination chemistry of other metals in the first transition metal series (e.g., Fe, cobalt, nickel, and Cu), Mn complexes have lower stability constants and are kinetically more labile [183]. The potential kinetic lability of Mn complexes is even more problematic than their lack of thermodynamic stability because of the possible Mn diffusion substitutions occurring with other ligands in vivo [184]. The lower stability constants of Mn complexes compared with other divalent transition metals and Zn(II) ions are attributed to the relatively large ionic radius of the Mn(II) ion and the configuration of its 3d electrons [185].

**FIGURE 2:**
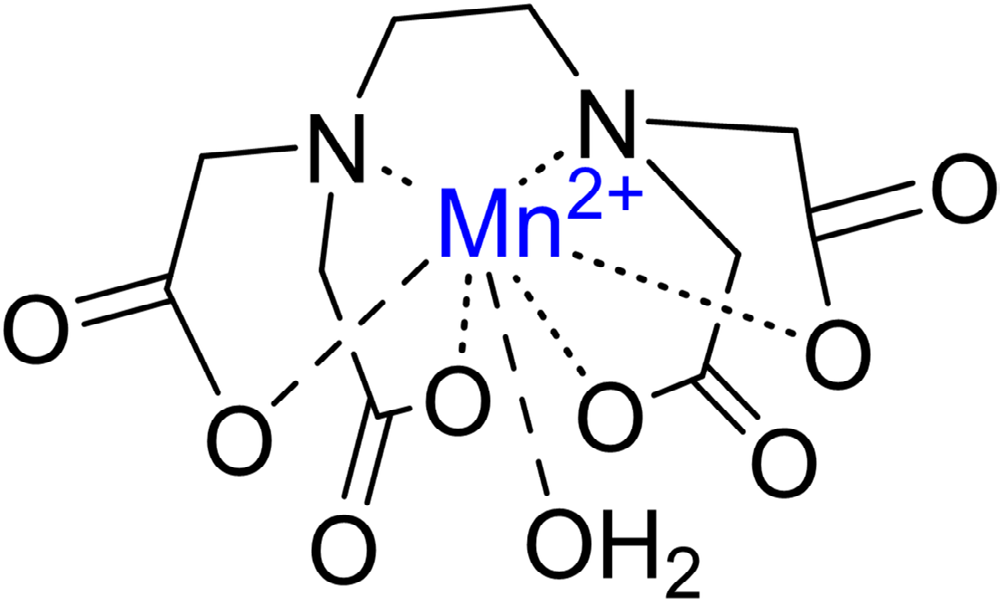
Structure of the complex formed between hexadentate EDTA and the manganous Mn^2+^ ion. A water molecule occupies a seventh coordination position in this complex ion [186, 187].

However, by building on the preference of Mn(II) for ligands which contain multiple *O*‐donors and *N*‐donors, for example ethylenediaminetetraacetic acid (EDTA, also known as edetic acid and versenic acid in the medical literature), a hexadentate ligand, and its salts, effective Mn coordination can be achieved (Figure 2).

Furthermore, a multi‐donor ligand framework is unable to assemble itself effectively around small metal ions, and it is better able to complex, for example, the larger Mn(II) ion, and offer metal selectivity. A survey of the coordination chemistry literature reveals that, at pH 7.4, some of the most stable Mn(II) complexes are formed from polyaminocarboxylate ligands, of which EDTA is the archetypal example [188].

### 3.1 Established Chelating Agents Which Have Been Investigated for the Treatment of Mn Overload

The following sections (summarized in Table 2) review chelating agents which have been used clinically or experimentally to date to treat Mn overload. All these agents had previously been investigated for other metal‐related disorders and, with the exception of EDTA, based on their chemical structures and weak coordination with Mn(II), have had little therapeutic impact in treating Mn‐related diseases [27, 172, 177, 191, 201, 205, 208]. The limited therapeutic efficacy of most of these ligands is attributed to the low stability constants of their Mn complexes and their kinetic lability, which allows facile ligand exchange and reduces complex stability in vivo.

**TABLE 2:** Current chelation treatment options for Mn overload.

Treatment	Chemical structure	Dosing	Side effects	Treatment efficacy
Disodium calcium edetate (Na_2_CaEDTA)	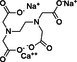	Intravenous, 20 mg/kg BD, monthly, for 5 days [27, 48, 107, 146, 165, 166, 189]	Nephrotoxicity, bone marrow suppression, Zn deficiency [6, 143, 190]	Clear evidence of clinical benefit: neurological improvement, halt of liver disease, correction of polycythaemia [145]
*meso‐*Dimercaptosuccinic acid (DMSA)	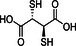	Intravenous or oral, 30 mg/kg/day for 3 days every 14 days [191]	Neutropenia, gastrointestinal (GI) discomfort, mucocutaneous changes [190, 192]	Some evidence of temporary clinical improvement: neurological and hepatic improvement [171, 193]
d‐penicillamine [194]	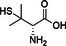	125–250 mg per day slowly increasing by 125–250 mg per week to 20 mg/kg per day in two divided doses (maximum 1500 mg/day) [172]	Cholestatic hepatotoxicity, immunoallergic manifestations (rash, fever, eosinophilia), bone marrow suppression, neutropenia, autoimmune conditions (glomerulonephritis, pneumonitis, lupus‐like syndrome), cutis laxa [158, 195, 196]	Some evidence of temporary clinical improvement: improvement of blood Mn [189]
Trientine (triethylenetetramine)	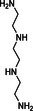	150–200 mg per day, slowly increasing by 150–200 mg per week to 400–1000 mg per day for trientine dihydrochloride (Cufence) or 225–600 mg per day for trientine tetrahydrochloride (Cuprior) in two divided doses [172]	Urticaria, other rashes, arthralgia, myalgia, proteinuria, hematuria, sideroblastic anaemia [172, 197]	Some evidence of temporary clinical improvement: correction of polycythaemia [174]
Sodium *p*‐aminosalicylic acid (PAS‐Na) and *N*‐acetyl‐*p*‐aminosalicylic acid (Ac‐PAS) [198]	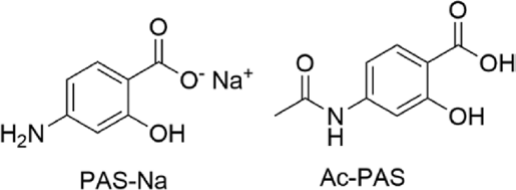	Oral, 6 g/day [177]	GI symptoms, skin reactions, rarely agranulocytosis [199]	Some evidence of temporary clinical improvement: neurological improvement of manganism [200]
Deferoxamine	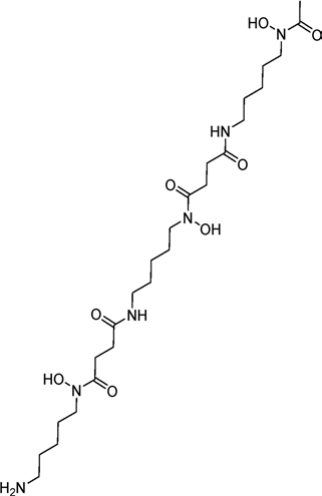	Subcutaneous or intravenous, 20‐60 mg/kg/day [201]	Injection‐site reactions, visual and auditory symptoms, bone‐growth retardation, intervertebral disc changes [202, 203].	Not used for Mn chelation in clinical settings [204]
Deferiprone	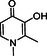	Oral, 75‐100 mg/kg/day [205]	GI symptoms, agranulocytosis, neutropenia, transaminitis [206]	Not used for Mn chelation in clinical settings
Deferasirox [207]	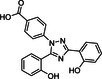	Oral, 5‐40 mg/kg/day [208]	Transaminitis, GI symptoms, skin rash, rise in creatinine [209, 210]	Not used for Mn chelation in clinical settings

#### 3.1.1 Na_2_CaEDTA (Disodium Calcium Edetate)

Na_2_CaEDTA is the most used chelating agent to treat Mn overload. Na_2_CaEDTA's Ca atom can be exchanged for metal ions that have a higher affinity for the chelating agent than Ca. This creates a water‐soluble compound that facilitates metal excretion via the kidney. Orally, EDTA is very poorly absorbed (< 5%) and thus requires parenteral administration [211]. Na_2_CaEDTA is not discriminant in its targeting of metals and chelates multiple metals such as Zn, Cu, Fe, Hg, and Cd [190]. Urinary Mn reaches its highest concentrations between one and 2 h after intravenous administration [212]. Prior to the identification of the inherited Mn transporter defects, Na_2_CaEDTA was mainly used to treat Pb poisoning [190, 213]. In chronic Mn poisoning due to environmental overexposure, Na_2_CaEDTA can lead to improvement of neurological symptoms, particularly the motor symptoms [214]. Repeat treatment cycles after removal from the exposure are likely to be required in order to efficiently remove Mn from the brain. In some cases, however, symptoms persist or progress [214, 215]. In both HMNDYT1 and HMNDYT2, patients can experience a dramatic improvement in their motor symptoms upon Na_2_CaEDTA chelation therapy, with some patients regaining the ability to walk [6, 27, 48, 147, 165, 166, 189, 216]. However, the response to Na_2_CaEDTA treatment can be variable, particularly in patients with HMNDYT2, with some patients showing minimal to no response in neurological symptoms and others experiencing worsening of dystonia [165, 167].

#### 3.1.2 Dimercaptosuccinic Acid (2,3‐Dimercaptobutanedioic Acid; DMSA)

The carboxyl and sulfhydryl sites of DMSA are both available for metal coordination. The *meso* form of DMSA (Succimer, INN) has usually been used therapeutically, but the superior water solubility of racemic DMSA offers advantages [217]. DMSA, an FDA‐approved compound, is used to treat Pb, Hg, and As overload [218, 219]. It can be administered intravenously, orally (20% absorption), transdermally, or rectally [157]. DMSA increases the excretion of Ag, Cd, Pb, and Hg via the urine while minimally affecting essential metals. Initial attempts to treat patients with occupational Mn intoxication did not show significant changes in blood and urine Mn levels [170]. However, patients who also received Fe supplementation in addition to DMSA showed amelioration in clinical symptoms. A similar response has been observed in patients with HMNDYT1 who received DMSA in combination with Fe supplementation, vitamin D, and C, and low levodopa doses. This combinatory treatment reduced Mn levels, improved liver function as well as ameliorated some neurological symptoms, with one patient regaining the ability to walk [171]. However, it is possible that this effect was primarily contributed by Fe supplementation [6]. Recent personal correspondence with Dr. Maha Zaki indicates that DMSA has not been effective in achieving long‐term reductions in Mn levels [171]. Multiple analogues with more improved chelating efficacy than DMSA have been made, such as monocyclohexyl DMSA (MchDMSA), monomethyl DMSA (MmDMSA), and monoisoamyl DMSA (MiADMSA), which may improve clinical response [156].

#### 3.1.3 D‐Penicillamine ((2*S* )‐2‐Amino‐3‐Mercapto‐3‐Methylbutanoic Acid)

D‐Penicillamine and trientine are the most commonly used chelating agents for the treatment of Cu overload in Wilson's disease patients. In the UK, clinicians tend to have more experience with D‐penicillamine [172]. D‐Penicillamine has three possible sites for metal coordination (amino, carboxyl, and sulfhydryl) and can therefore act as a monodentate, bidentate, or tridentate ligand [220]. Administered orally, D‐penicillamine is usually effective for treating Wilson's disease, although 20%–30% of patients can experience an exacerbation of neurological sequelae in the initiation phase of treatment [221]. Secondary or tertiary treatment options for Pb and As toxicity may also include D‐penicillamine [222, 223]. There is limited data on the Mn chelating efficacy of D‐penicillamine. While urinary excretion of Mn only minimally increases compared to Na_2_CaEDTA, some favorable effect of D‐penicillamine has been observed in patients with HMNDYT1 who could not remain on Na_2_CaEDTA therapy [89]. Blood Mn levels decreased and one patient showed an improvement in dystonia [189]. D‐Penicillamine also appears to have an additive effect when administered in conjunction with Na_2_CaEDTA, leading to further clinical improvement [165].

#### 3.1.4 Trientine (Triethylenetetramine)

Triethylenetetramine, a tetradentate ligand, coordinates metals through its four amino groups and is particularly effective for complexing Cu(II) ions [224, 225, 226]. Trientine dihydrochloride was introduced in 1969 for Wilson's disease patients intolerant of D‐penicillamine [227, 228]. More recently, trientine tetrahydrochloride has been introduced and subsequently given FDA approval for the treatment of Wilson's disease [229]. Trientine has been used successfully in one patient with acquired hepatocerebral degeneration who presented with tremor, bradykinesia, and rigidity. Blood Mn was raised five times that of normal with apparent Mn deposition on brain MRI. After 4 months of treatment with trientine, blood Mn levels normalized, and tremor, Parkinsonism, and brain Mn deposition reduced markedly [173]. Trientine, in conjunction with Fe supplementation, was also used in a patient with HMNDYT1 [174]. Mn induced polycythaemia resolved, and Mn deposition reduced as evidenced by brain MRI. Again, whether the reduction in Mn load can be primarily attributed to trientine treatment or is solely the effect of Fe replacement requires further validation since Fe supplementation alone can dramatically improve Mn levels and neurological symptoms in SLC30A10 deficiency [179].

#### 3.1.5 Sodium *p*‐Aminosalicylic Acid (4‐Amino‐2‐Hydroxybenzoic Acid, Sodium Salt) and *N* ‐Acetyl‐ *p* ‐Aminosalicylic Acid

Sodium *p*‐aminosalicylic acid (4‐amino‐2‐hydroxybenzoic acid, sodium salt) (PAS‐Na) was developed in the 1940s as an antituberculosis drug [230]. Mn‐PAS complexes MnL and MnL_2_ (L = PAS) have been characterized with Mn coordinated to the hydroxyl and carboxyl groups of PAS [198, 231, 232]. Multiple studies in rats have shown that PAS‐Na enables the elimination of Mn from the brain and the liver [233, 234, 235]. Additionally, PAS‐Na can effectively ameliorate Mn‐induced inflammation, oxidative stress, and DNA damage in neuronal and microglial cultures and rats [236, 237, 238]. PAS‐Na may therefore have dual effects on Mn neurotoxicity via chelation as well as antioxidant function.

Clinical effectiveness of PAS‐Na in treating Mn overload has been demonstrated in patients suffering from chronic Mn poisoning, leading to improvement of fine motor co‐ordination and normalization of brain MRI [175]. PAS‐Na in combination with Na_2_CaEDTA has been shown to normalize brain MRI appearances and improve gait and tremor in patients with manganism [176]. There is limited clinical experience with PAS‐Na in inherited Mn transporter defects HMNDYT1 and HMNDYT2. One patient with HMNDYT1 did not appear to show any neurological improvement upon PAS‐Na treatment [239].

PAS's *N‐*acetylated metabolite (Ac‐PAS) binds more strongly than PAS to Mn(II) [231]. Both PAS‐Na and Ac‐PAS can cross the blood–brain barrier and mobilise Mn from the brain in Mn exposed animals [231, 240, 241, 242]. Thus, in cases of patients suffering from Mn overload, PAS‐Na could serve a twofold effect in chelating the excess Mn as well as ameliorating Mn‐induced neurotoxicity effects [200]. The potential to develop new chelating agents based on PAS and its major metabolite holds some promise [243].

#### 3.1.6 Deferoxamine, Deferiprone and Deferasirox

Deferoxamine (DFO) as well as the newer, orally bioavailable iron chelating agents deferiprone (DFP) and deferasirox (DFX) have a higher affinity and form stronger bonds with trivalent ions. Thus, they are utilized in diseases that result in Fe overload including transfusion dependent thalassemia and sickle cell anaemia, and haemochromatosis [156, 244]. Studies in male Wistar rats demonstrated that each Fe chelating agent alone or in combination effectively reduces Mn levels in the blood and brain in environmental Mn overload [245, 246]. However, no reports of using DFO, DFP, or DFX in clinical settings of Mn overload are available.

## 4 Development of Novel Mn Chelators

**FIGURE 3:**
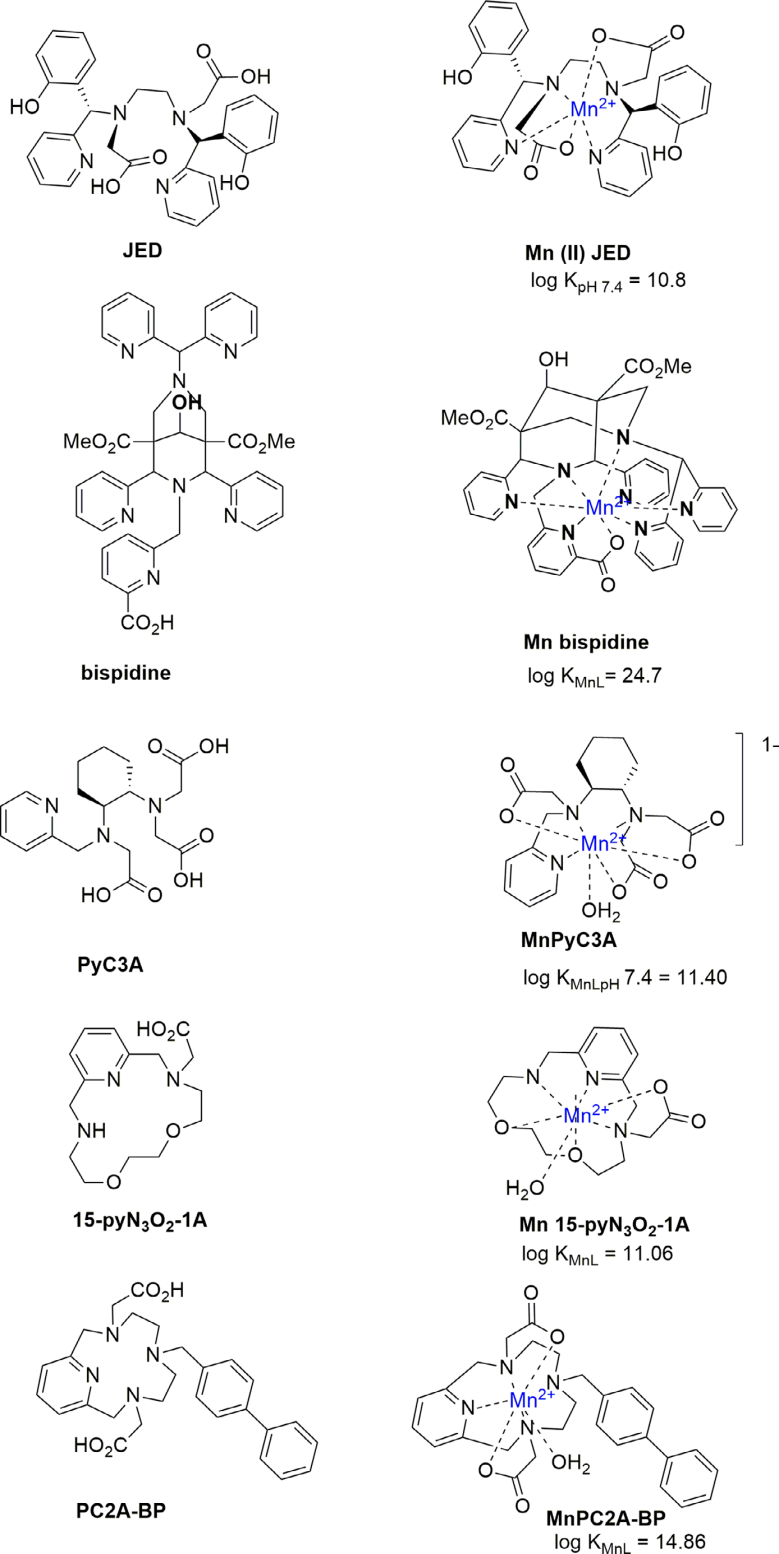
Structures of novel Mn‐coordinating ligands suggested for use as Mn MRI contrast agents [184, 187, 247, 248, 249, 250, 251, 252, 253, 254, 255]. Stability constant data show the effectiveness of these ligands for coordinating Mn. Measurement of these constants for each individual ligand would have been carried out under slightly different conditions (pH, temperature, and ionic strength—see the references for experimental details); hence, these stability constants, though illustrative of improvements in ligand design for coordinating Mn, are not directly comparable.

Novel Mn‐coordinating ligands have been developed in recent years as potential alternatives to MRI contrast agents that currently contain the rare earth metal gadolinium (as Gd(III)). The design of these ligands, which form thermodynamically and kinetically stable complexes with Mn, has gained momentum due to the growing need for safer contrast agents that could replace Gd(III)‐based compounds in clinical practice [247]. Repurposing these ligands as Mn chelators represents a promising strategy for the development of new treatments targeting conditions associated with Mn accumulation. The Mn(II) ion exhibits all the physical properties that make the Gd(III) ion a highly efficient imaging probe in MRI. However, the substitution of Mn for Gd in MRI contrast agents is not without challenges, namely, the lack of ligand field stabilization in the high spin d^5^ electronic configuration of Mn(II), a property which induces lower thermodynamic stability in Mn complexes, and the need (in contrast agents) for an inner sphere coordinated water molecule. Many groups have resorted to macrocyclic (large ring) ligands and rigid linear ligands in an attempt to stabilize Mn complexes for use as contrast agents, and some examples of these ligands are shown in Figure 3 [187]. The “Janus” ligand, JED, which allows a switch between Mn(II) and Mn(III), offers the option of Mn coordination between these two oxidation states [187]. The Mn complex of bispidine was stable for over 140 days at pH 6 at 37°C, even in the presence of 50 equivalents of Zn(II) [248, 249]. PyC3A forms a stable Mn(II) complex at physiological pH, whereas the Mn complex of the macrocycle 15‐pyN_3_O_2_‐1A is not suitable for in vivo application due to its rapid dissociation (loss of Mn) [247, 250, 251]. The Mn complex of PC2A‐BP is not only kinetically inert (*t*_1/2_ at pH 7.4 is 286.2 h) and thermodynamically stable but also has a hydrophobic tail capable of engaging human serum albumin, which increases its half‐ life [252].

The new ligands designed for these complexes feature some or all of the requirements for effective and selective Mn coordination discussed above: multidentate N or N and O ligands and rigid linear or cyclic cavities large enough to contain Mn(II) but unsuitable to hold smaller ions. Thus, the new Mn contrast agents have structural features better designed to bind Mn, and these are absent from the ‘older’ generation of Mn ligands shown in Table 2. The application of these new ligands for Mn chelation therapy is therefore a possibility. However, despite our better understanding of how to effectively coordinate Mn, there remains an unmet clinical need to discover new synthetically accessible drugs with the desired physiochemical attributes that can be administered orally for Mn overload disorders.

## 5 Alternative Therapies

While some progress has been made in the development of effective Mn chelation strategies, it is essential to also consider the potential of genetic therapies for treating inherited disorders such as HMNDYT1 and 2. Substantial advances in gene and mRNA therapies have been achieved in recent years, and it is likely that such approaches will be developed for a growing number of rare genetic diseases in the future [256, 257, 258]. Although these therapies target the underlying genetic defect, concerns remain regarding immune responses, insertional mutagenesis, off‐target effects, long‐term safety, and significant socioeconomic costs [259, 260, 261]. Chelation therapy will continue to play an important complementary role, as it can delay or prevent Mn accumulation prior to the administration of genetic therapies and help mitigate disease progression during the interim.

## 6 Current Consensus on the Management of Mn Overload

With reference to the recently published consensus recommendations led by Dr. Karin Tuschl and an international expert group, current evidence indicates that Na_2_CaEDTA remains the chelating agent of choice for treating Mn overload [145]. Although its administration regimen is burdensome, there is clinical evidence that monthly intravenous Na_2_CaEDTA reduces Mn accumulation in the brain and liver, which is associated with neurological improvement, stabilization of liver disease, and correction of polycythaemia [6, 27, 48, 63, 146, 147, 149, 152, 165, 166, 169, 174, 189, 262, 263, 264]. For HMNDYT1, which is characterized by iron depletion alongside hypermanganesaemia, iron supplementation can help reduce Mn accumulation, either as monotherapy or in combination with other chelating agents [89, 179, 265].

Other Mn chelators trialled in patients with HMNDYT1 or HMNDYT2 include DMSA, D‐Penicillamine, trientine, and PAS. While some benefit has been reported when these agents are combined with iron supplementation or Na_2_ CaEDTA, none has demonstrated sustained clinical efficacy when used alone [165, 171, 174, 189, 239].

## 7 Conclusions

The significance of Mn in neurodegenerative disease processes is becoming more evident in the context of both the rare inherited Mn transporter defects as well as common neurodegenerative disorders, with Mn playing an important role as an environmental toxicant. Therefore, efficient and safe Mn chelation is a priority for multiple disease entities to prevent Mn‐associated neurotoxicity. Chelators currently used in the clinic are inadequate due to their side effect profile and need for extensive hospitalization, as well as their lack of metal specificity. The discovery of novel Mn‐specific chelators derived from Mn‐based MRI contrast agents provides a promising avenue to develop orally bioavailable chelating agents. Therapeutic optimization of novel chelators via combinatorial use or in conjunction with Fe supplementation may further enhance Mn elimination. Whilst developing new chelating agents is essential, targeting the mechanisms that elicit Mn overload in inherited Mn transporter defects, such as HMNDYT1 and HMNDYT2, through gene or mRNA therapies is an alternative therapeutic avenue. Using these therapies in conjunction with chelation therapy will ensure maximal potential for prevention and recovery of symptoms in affected patients.

## Author Contributions

All authors conceived the manuscript. H.V. and K.T. wrote the manuscript. G.E.K., R.P., and J.S. substantially revised and approved the final version.

## Consent

The authors have nothing to report.

## Conflicts of Interest

The authors declare no conflicts of interest.

## Data Availability

Data sharing is not applicable to this article as no new data were created or analyzed in this study.
